# Prenatal development is linked to bronchial reactivity: epidemiological and animal model evidence

**DOI:** 10.1038/srep04705

**Published:** 2014-04-17

**Authors:** Katharine C. Pike, Shelley A. Davis, Samuel A. Collins, Jane S. A. Lucas, Hazel M. Inskip, Susan J. Wilson, Elin R. Thomas, Harris A. Wain, Piia H. M. Keskiväli-Bond, Cyrus Cooper, Keith M. Godfrey, Christopher Torrens, Graham Roberts, John W. Holloway

**Affiliations:** 1 Clinical and Experimental Sciences Academic Unit, University of Southampton Faculty of Medicine, Southampton, UK; 2 Human Developmental and Health Academic Unit, University of Southampton Faculty of Medicine, Southampton, UK; 3 NIHR Southampton Respiratory Biomedical Research Unit; 4 NIHR Southampton Biomedical Research Centre, University of Southampton and University Hospital Southampton NHS Foundation Trust, Southampton, UK; 5 Medical Research Council Lifecourse Epidemiology Unit, University of Southampton, Southampton, UK; 6 These authors contributed equally to this work.; 7 These authors jointly directed this work.

## Abstract

Chronic cardiorespiratory disease is associated with low birthweight suggesting the importance of the developmental environment. Prenatal factors affecting fetal growth are believed important, but the underlying mechanisms are unknown. The influence of developmental programming on bronchial hyperreactivity is investigated in an animal model and evidence for comparable associations is sought in humans. Pregnant Wistar rats were fed either control or protein-restricted diets throughout pregnancy. Bronchoconstrictor responses were recorded from offspring bronchial segments. Morphometric analysis of paraffin-embedded lung sections was conducted. In a human mother-child cohort ultrasound measurements of fetal growth were related to bronchial hyperreactivity, measured at age six years using methacholine. Protein-restricted rats' offspring demonstrated greater bronchoconstriction than controls. Airway structure was not altered. Children with lesser abdominal circumference growth during 11–19 weeks' gestation had greater bronchial hyperreactivity than those with more rapid abdominal growth. Imbalanced maternal nutrition during pregnancy results in offspring bronchial hyperreactivity. Prenatal environmental influences might play a comparable role in humans.

Under the ‘developmental origins of health and disease’ hypothesis physiological and anatomical changes invoked by early environmental factors are able to influence later health. This is sometimes referred to as ‘programming’ or ‘developmental induction’^1^. Factors capable of invoking developmental induction before birth include maternal diet^2^, body composition^3^ and endocrine status^4^. Epidemiological evidence suggests that faltering fetal growth is associated with adverse respiratory outcomes. Following from early observations of increased chronic obstructive pulmonary disease in adults who were of low birthweight^5^, studies linking faltering fetal growth to wheeze in childhood have provided further evidence that early environmental factors can influence respiratory development^6^,^7^. These epidemiological observations do not provide information about underlying pathophysiological mechanisms; to understand these, animal models of fetal growth restriction are required^8^. Moreover, these models should reflect that whilst the majority of of human infants in westernized countries are of normal weight at birth, adverse consequences may occur as a result of growth faltering during a critical window of development.

Animal data demonstrate that maternal protein restriction in rats results in hypertension in the offspring^9^. This is linked to clinical evidence that aortic compliance is lower in adults born at low birthweight^10^ and that low birthweight individuals have an increased likelihood in adulthood of cardiovascular disease, including hypertension^11^. In part, the association between early growth restriction and hypertension may reflect adaptive changes affecting vascular smooth muscle. Animal and epidemiological studies suggest that bronchial smooth muscle might be similarly sensitive to environmental influences. Hyperreactivity of bronchial smooth muscle has been demonstrated in individuals born at low birthweight (a surrogate for restricted fetal growth)^12^,^13^. Animal models also show bronchial hyperreactivity (BHR) to be present in mice exposed to adverse environmental factors which are likely to restrict fetal growth, for example maternal stress^14^. We hypothesize that an adverse *in utero* environment, in this case imbalanced nutrition, is associated with BHR in the offspring. The primary objective of the animal work included in this study was to investigate this using a model which has already demonstrated a number of the cardiovascular risk factors associated with poor fetal growth (including hypertension and endothelial dysfunction)^15^,^16^, Since Rho A has been implicated in bronchial hyper-responsiveness in mouse models^17^, rat models^18^ and humans^19^,^20^ a secondary objective was to use the animal model to explore whether Rho A, and associated kinases ROCK1 and ROCK2 may be sensitive to developmental stress and hence serve as a link between adverse factors in the fetal environment and later BHR. Finally, to test the relevance of factors affecting fetal growth to human respiratory development, we analysed data from an epidemiological cohort where both detailed prenatal ultrasound measurements and childhood BHR measurements are available.

## Results

### Animal model

#### Birthweight and growth

There were no between group differences in litter size or birthweight. This was true for both male (C, 7.60 g ± 0.16, n = 7; PR, 7.74 g ± 0.40, n = 6; P > 0.05) and female (C, 7.41 g ± 0.19, n = 7; PR, 7.46 g ± 0.34, n = 6; P > 0.05) pups. Similarly growth of the offspring was not different between the groups (data not shown) and there was no significant difference between the rats at 75 days of age (C, 320.6 g; PR, 280.4 g; p > 0.05).

#### Bronchoconstriction

**Figure 1:**
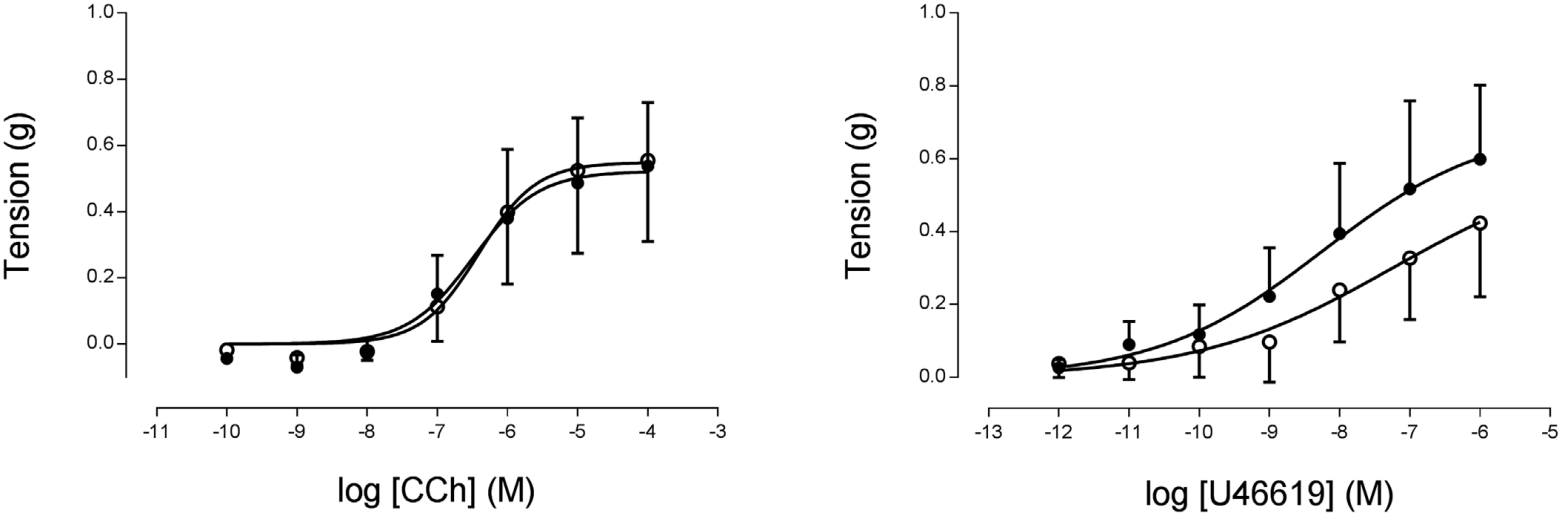
Cumulative addition of (A) CCh or (B) the thromboxane mimetic U46619 to isolated bronchi from 35 day old male offspring from C (o, n = 6) or PR (•, n = 5) dams.

**Figure 2:**
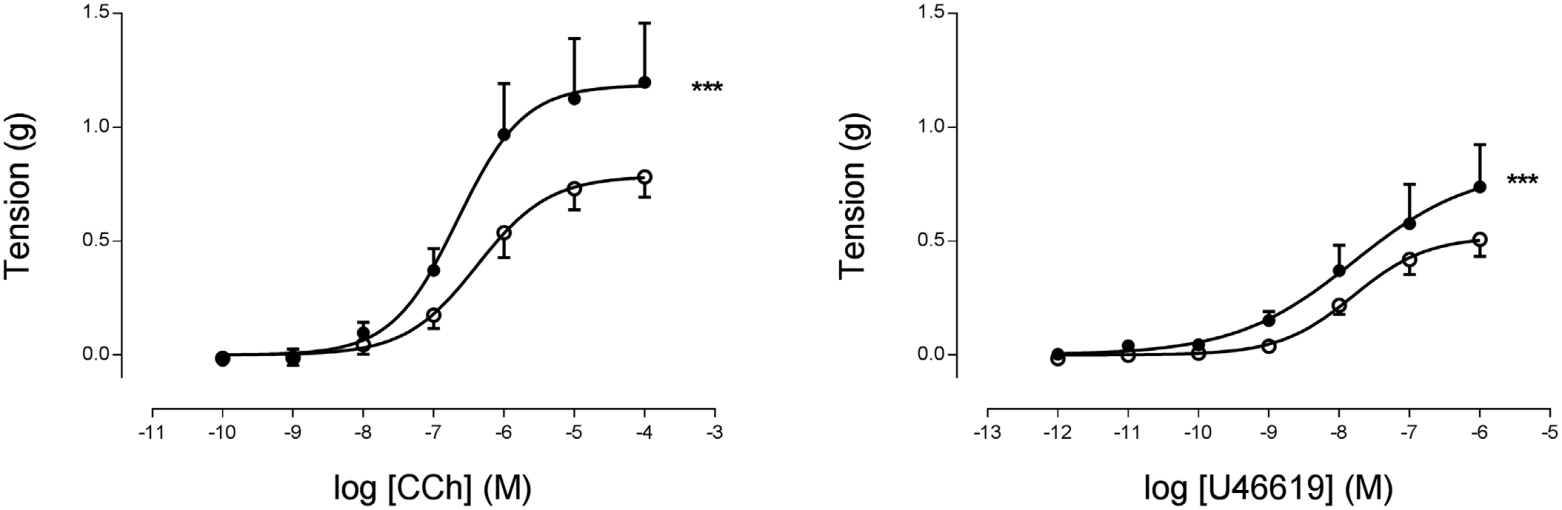
Cumulative addition of (A) CCh or (B) U46619 to isolated bronchi from 75 day old male offspring from C (o, n = 6) or PR (•, n = 4) dams. *** indicates p < 0.001% max response C vs. PR.

In isolated bronchi from 35-day-old rats, both CCh and U46619 produced a concentration-dependent constriction that was similar between groups (p > 0.05; Figure 1). By 75 days of age maximal response to both agonists was significantly increased in the PR bronchi compared to controls (*p* < 0.001; Figure 2). In the presence of the Rho kinase inhibitor Y27632, differences in response to carbachol were abolished (Figure S1). Responses to the depolarising KPSS wash were similar between the groups at both time points (data not shown).

#### RhoA, ROCK1 and ROCK2 mRNA expression levels

In isolated bronchi from 75-day-old male offspring, mRNA levels of Rho A were similar between the groups (C, 0.59 ± 0.03, n = 5; PR, 0.60 ± 0.02, n = 4; P > 0.05). Equally, mRNA levels of ROCK1 (C, 0.62 ± 0.11, n = 5; PR, 0.65 ± 0.05, n = 4; P > 0.05) and ROCK2 (C, 0.78 ± 0.04, n = 5; PR, 0.94 ± 0.13, n = 4; P > 0.05) were not different between the two groups.

#### Morphometry

**Figure 3:**
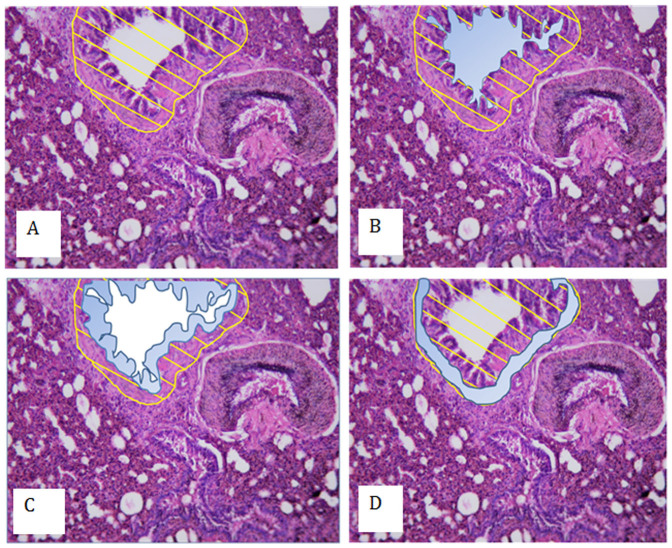
Comparison of airway wall histology in offspring of control and protein restricted mothers.

**Figure 4:**
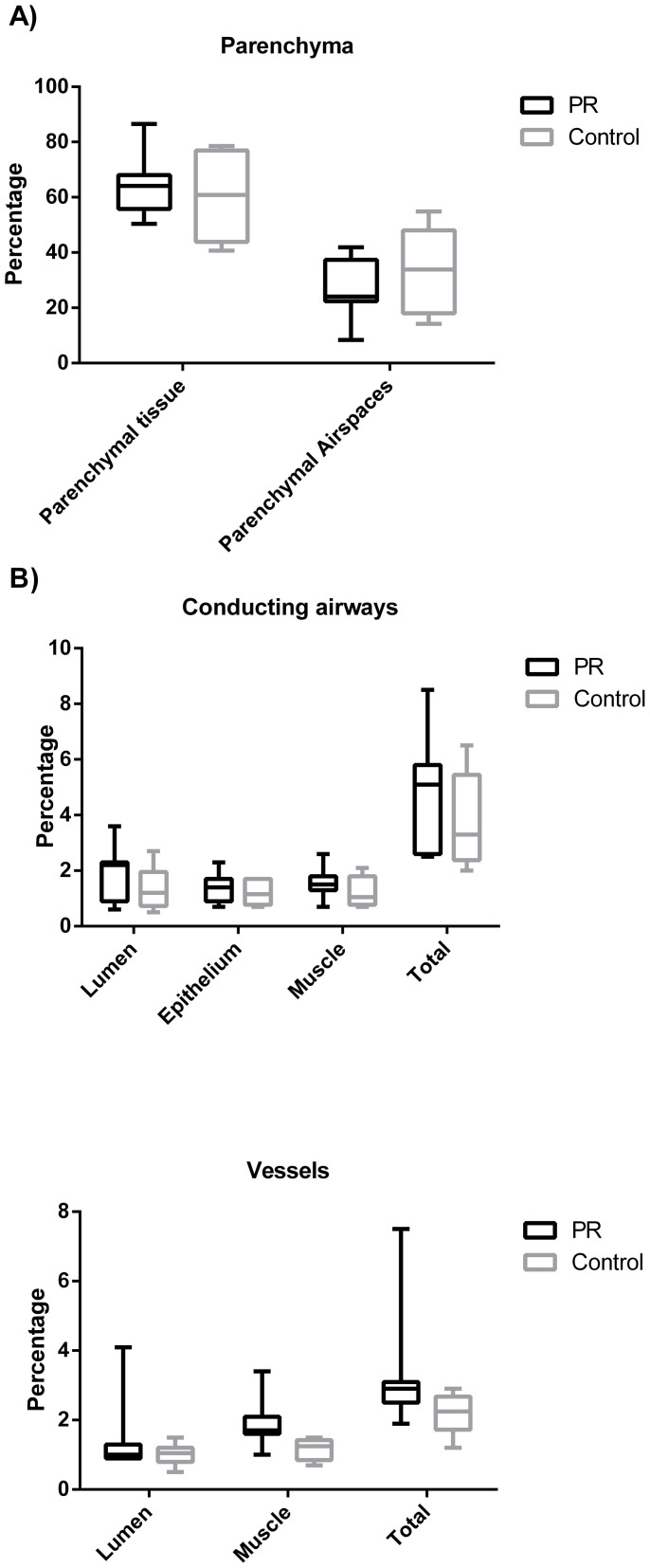
Volume factionation of lung components as percentage of entire lung for both PR and C groups at 225 days of age (C = 6, PR = 6).

There was no between group difference in percentage airway smooth muscle at day 225. Equally, no between group differences were seen for percentage of any other airway component (lumen or epithelium), percentage parenchymal tissue or airspaces, or vessel lumen. The volume fraction of vascular muscle was significantly greater in the protein restricted group compared to control (median 1.70 vs. 1.25, p = 0.01) (Figures 3 and 4).

### Fetal growth and BHR in childhood

Mothers and children followed up to 6 years were broadly similar in terms of asthma, atopy and allergic disorders to those who were not followed up. Participant mothers were, however, of lower parity and were slightly older, taller, less likely to smoke in pregnancy and were of higher educational attainment than non-participants (Table S2). Of the participating mother-child pairs, those who provided methacholine provocation challenge data had a significantly higher number of siblings than those who did not complete a methacholine challenge but were otherwise comparable (Table S3). Maternal smoking during pregnancy was not found to be a confounder of the relationship between fetal growth and BHR.

#### Methacholine provocation challenge

Higher inverse log values of the dose response slope, (lower BHR), were significantly associated with increasing conditional abdominal circumference growth between 11 and 19 weeks' gestation (p = 0.037). That is to say, greater faltering of abdominal growth was associated with greater BHR. There were no significant associations between birthweight and BHR. Moreover, no association was found between abdominal circumference growth between 19 and 34 weeks' gestation, or birth head or abdominal circumference and BHR (Table 1).

## Discussion

This paper demonstrates, for the first time, BHR following maternal dietary restriction in an animal model. This is supported by observations in a human population birth-cohort. In the offspring of protein-restricted rats, bronchoconstriction to both CCh and U46619 was significantly enhanced compared to control animals; this phenotype was not due to altered airway structure. We also demonstrated an inverse association between abdominal circumference growth between 11 and 19 weeks' gestation and BHR measured in 6-year-old children. These findings suggest that unfavourable *in utero* conditions might adversely affect respiratory development and perhaps predispose to subsequent respiratory disease.

This study shows that restricted fetal growth is associated with BHR in both rats and humans. Our data also suggest that airway smooth muscle changes may underlie this association. These are novel animal model findings that are supported by the Southampton Women's Survey, one of few epidemiological cohorts with detailed longitudinal ultrasound scan measurements of fetal growth. Reduced abdominal growth is recognized as an important fetal adaptation to *in utero* adversity, which is believed to protect brain growth^27^. Notably there was no association between infant birthweight and BHR in childhood; the association found between reduced abdominal growth in early pregnancy and BHR shows similarities to the findings in the animal model and suggests common ‘programming’ mechanisms, possibly mediated via subtly altered fetal growth patterns acting in both animals and humans subjected to developmental stress. The significant relationship between abdominal circumference growth in early pregnancy and BHR may reflect a period of particular importance in bronchial smooth muscle development. Recently, Zaina-Taieb *et al* have used a very similar protocol to ours in order to study the development of bronchopulmonary dysplasia. Their study looked at early morphometry with differences in alveolarisation, whereas our study found no difference at 28 or 225 d. Interestingly, Zana-Taieb *et al* showed significant differences in birthweight, such that the protein restricted group could be considered IUGR. Previous studies have also linked IUGR to changes in lung structure whilst our study produced normal sized offspring with hyper-reactive airways. It is not clear how their protocol produced IUGR offspring whilst ours did not.

The significance of early growth restriction in relation to respiratory health has been appreciated since the report of associations between infant weight and death in adulthood from respiratory disease^5^. Moreover, impaired lung function has been found in individuals who were of low birthweight^28^,^29^,^30^. Reduced forced expiratory flows have been demonstrated in lower birthweight individuals within a group of term babies of normal average birthweight; the lowest lung function was found in infants who gained weight rapidly after birth^31^. This pattern of growth may indicate mismatched pre- and postnatal nutrient supply and thus identify infants subjected to fetal growth restriction. Within the same cohort lower infant lung function has been shown to be associated with wheezing illnesses in early childhood^32^. Taken together with epidemiological evidence showing lower birthweight to be associated with later respiratory morbidity, including asthma^33^ and chronic obstructive pulmonary disease^5^, this suggests lung function may be persistently impaired following fetal growth faltering and supports the concept of lung function ‘tracking’ throughout life^34^.

An association between low birthweight and BHR has been recognized for some time^35^ but studies addressing the effect of fetal growth restriction upon this specific aspect of lung function have been confounded by the effects of respiratory complications of prematurity and their treatment^36^,^37^. Although there is a wealth of evidence on low birthweight and respiratory health^38^,^39^, there is a relative paucity of data relating these outcomes to fetal growth within those who are not classified as growth restricted. Offspring from our animal study are of normal birthweight but were exposed to nutrient stress during prenatal development, similarly our human data include infants whose birth weights were within the normal range but whose growth, possibly due to adverse environmental influences, faltered during a critical developmental window. This may be highly relevant to understanding the prenatal origins of respiratory disease in the developed world where most children are born well grown but some have suffered a stress during a critical developmental window. Four studies have explored the relationship between ultrasound measures of fetal size and wheeze; of these, three found an association between restricted fetal growth and childhood wheeze^6^,^7^,^40^. Recently a fourth study failed to find any such association^41^, although this study did not assess equivalent dimensions at each gestation. Twin studies, which study the effects of fetal growth restriction independently from shared gestation, have demonstrated greater BHR to cold air challenge in the smaller twin of twin pairs discordant for birthweight^13^. In the present study fetal growth was directly assessed by longitudinal ultrasound of equivalent dimensions and confounding by neonatal respiratory disease or treatment is unlikely as participants were term subjects of normal birthweight.

Animal data suggest that adverse prenatal factors which are likely to affect fetal growth are associated with increased BHR. For example, increased BHR has been found in offspring of mice exposed to noise stress during pregnancy^14^. Similarly, we demonstrate that isocaloric reduction of maternal protein intake in rats leads to BHR in the offspring. BHR is thought to arise from an interplay between airway remodelling, inflammatory processes and altered smooth muscle^18^,^42^,^43^,^44^, however the exaggerated bronchial responsiveness in our model was not associated with gross airway remodelling. This suggests that BHR precedes bronchial remodelling, that remodelling is not a prerequisite for BHR development and that developmental factors may alter intrinsic smooth muscle function. Recently it has been shown that chronic airway smooth muscle stimulation can lead to muscle shortening and greater constriction to subsequent challenges^45^, while airway remodelling has been reported in response to a methacholine challenge alone^46^. Taken together this suggests repeated constrictions may predispose to remodelling in their own right.

A possible explanation for the exaggerated constriction in the present study is altered signalling in the bronchial smooth muscle contractile apparatus. Maternal nutrient restriction in sheep leads to excessive vasoconstriction in specific arterial beds of the offspring^47^,^48^,^49^ which is associated with an increase in myosin light chain kinase mRNA levels^47^,^48^. An increase in contractile apparatus could explain our results, but such increases would also have manifested in an increased contraction to a depolarising K^+^ wash. This did not occur, and enhanced constriction was only seen in response to agonists, suggesting a role for Rho A and the Ca^2+^-independent pathway. Rho A and its downstream target Rho-associated kinase enhance constriction by inactivating myosin light chain phosphatase^50^. Previous reports have demonstrated that inhibition of Rho associated kinase leads to decreased bronchial contraction^51^, a finding which we have confirmed in the present study. Crucially, however, we have shown that the exaggerated response to CCh seen in the PR group is no longer seen in the presence of the Rho kinase inhibitor, suggesting a key role for Rho A in the BHR induced by maternal protein restriction. At present it is unclear why increased bronchial responsiveness was seen in the 75-day-old but not the 35-day-old rats, although potentially an age-dependent change in Rho A or Ca^2+^-dependent signalling may explain this. Other studies have specifically implicated translocation of Rho A to the membrane in the development of BHR^18^. The importance of this post-translational modification might also explain why we detected no difference between experimental groups in Rho A mRNA. Further investigation within this animal model would support exploration of the mechanisms believed to underlie increased BHR, whilst in vivo animal model testing would confirm the relevance of the model to human disease.

In summary, *in utero* stress conferred by protein restriction during pregnancy increases bronchial reactivity in rats and there are epidemiological data that suggest a similar effect might exist in humans. Taken together these findings suggest adverse *in utero* conditions are associated with a specific impairment of respiratory development.

## Methods

### Ethics statement

All animal procedures carried out in this study were in accordance with the regulations of the British Home Office Animals (Scientific Procedures) Act, 1986 (under licence number PPL 30/2884) and this study was approved by the local ethical review committee. Animals were sacrificed by cervical dislocation. For human participants written informed parental consent was obtained and ethical approval for this and all other human aspects of the study was granted by the Southampton and South West Hampshire Research Ethics Committee (276/97, 307/97, 089/99, 06/Q1702/104).

### Animal model

Virgin female Wistar rats (Harlan, UK) were mated with stud males. After confirming conception mothers were fed either a control (C; 18% casein) or a protein-restricted diet (PR; 9% casein)^21^. Diets were isocaloric and of comparable vitamin and mineral content^16^. Mothers and pups were fed standard laboratory chow postpartum.

Pups were weighed at 48 hours and litters culled to eight. Weaning was at 21 days. At 35, 75 or 225 days of age male offspring were sacrificed and lung tissue harvested.

#### Assessment of bronchoconstriction

Lungs were excised and placed in 4°C physiological salt solution (PSS), (NaCl, 119; KCl, 4.7; CaCl_2_, 2.5; MgSO_4_, 1.17; NaHCO_3_, 25; KH_2_PO_4_, 1.18; EDTA, 0.026; and D-glucose, 5.5 mM). Third generation bronchi were dissected into 2 mm segments and mounted on a wire myograph (Danish Myo Technology A/S, DK). Segments were maintained in 37°C PSS, gassed with 95% O_2_ and 5% CO_2_.

Segments were stretched to optimal resting tension (1.5 g) and allowed one hour equilibration. Functional integrity was tested using 125 mM KPSS solution (PSS with equimolar KCl for NaCl substitution). Cumulative concentration-response curves were constructed for the acetylcholine mimetic, carbachol (Sigma-Aldrich®) (CCh, 1 nM–10 μM) and the thromboxane mimetic, U46619 (Tocris Bioscience) (1 pM–1 μM). In a subgroup, CCh responses were repeated in the presence of the Rho kinase inhibitor Y27632 (Sigma-Aldrich; 10 μM).

#### Molecular biology

mRNA expression in isolated bronchi was determined using real time PCR relative to β-actin (TaqMan, Applied Biosystems, Warrington, U.K.). Primers and probes are given in Table S3.

#### Morphometry

Left lungs were formalin fixed for 24 hours and embedded in Paraffin wax. An unbiased stereological analysis approach was employed^22^. Starting at a random point a 5 um section was cut 500 μm intervals throughout the entire lung. Sections were H&E stained, 30–70 photographs taken of each section and 1 in 10 analysed to determine the volume fraction of airway tissue, airway lumen, epithelium, smooth muscle using a point counting system (Figure 4).

### Human participants

**Figure 5:**
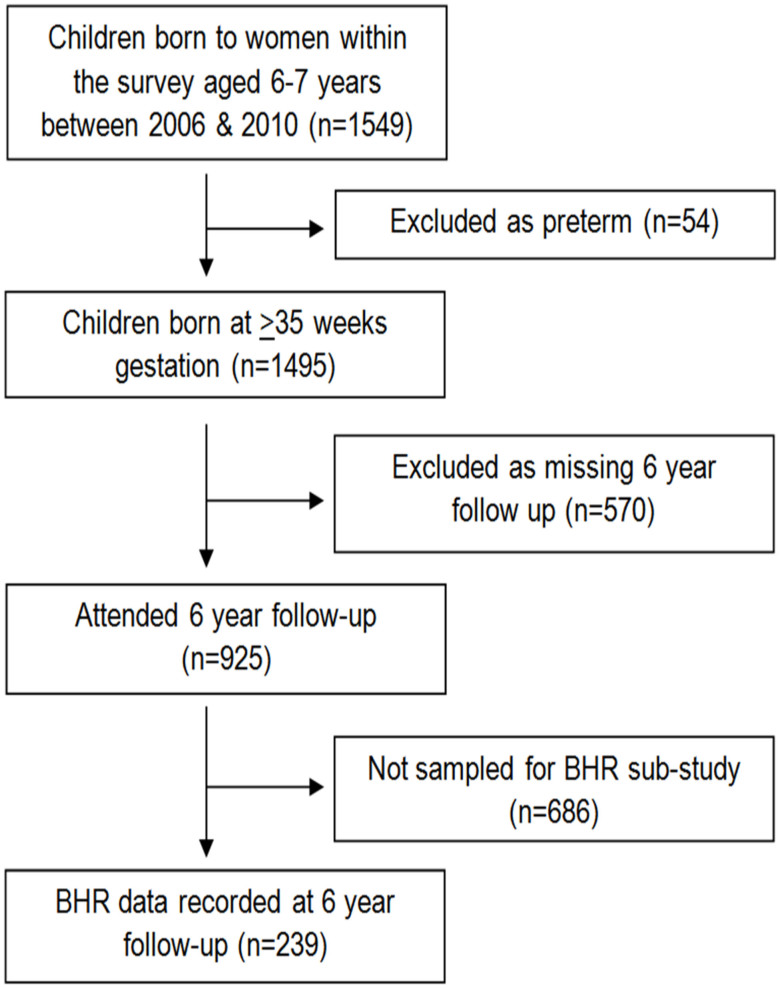
Follow-up within the Southampton Women's Survey.

Participants were mother-child pairs from the Southampton Women's Survey^23^. Six-year-old children were invited for respiratory follow-up; 246 underwent methacholine challenge (Figure 5). Seven children born < 35 weeks' gestation were excluded to remove the effects of prematurity.

#### Fetal growth

Gestational age was determined from last menstrual period and early ultrasound data. Fetal head and abdominal circumferences at 11, 19 and 34 weeks' gestation were measured according to standardized landmarks using Acuson 128 XP, Aspen and Sequoia ultrasound machines calibrated to 1540 m/s. Head and abdominal circumferences and weight were measured at birth. Conditional velocities of prenatal head and abdominal circumference growth were calculated, correcting for age at measurement and regression to the mean^24^.

#### Bronchial hyperreactivity

Bronchial hyperreactivity was measured by bronchial provocation challenge, according to ATS/ERS guidelines^25^. Incremental methacholine concentrations (0.06 mg/ml to 16 mg/ml) were delivered using a dosimeter (Koko; PDS Instrumentation; Louisville, USA) and a compressed air driven nebulizer (Sidestream®; Respironics, UK). Challenges were terminated following a 20% fall in the FEV_1_ or, if this did not occur, following the 16 mg/ml dose. BHR was expressed as the inverse of the slope of the regression line through FEV_1_ drop and logged methacholine concentration such that lower inverse log slope values indicate increased BHR.

Log slope = 100/[regression slope of FEV_1_ drop and log_10_(cumulative methacholine dose) + 10].

A constant removes negative values and an inverse transformation ensures the variable is normally distributed^26^.

### Statistical analysis

Animal data are mean ± (S.E.M) and constrictor responses are change in raw tension (g) or percentage KPSS-induced tone. Cumulative response curves were analysed by fitting a four-parameter logistic equation using non-linear regression to obtain pEC_50_ (effective concentration equal to 50% of maximum) and maximal response which were compared by Student's *t* test (Prism 5.0, GraphPAD software Inc., San Diego, CA, U.S.A.). Significance was accepted if *p* < 0.05. The investigator was blinded to dietary group. Lung structures were expressed as percentage of total lung and compared using the Mann Whitney rank sum test.

Childhood BHR was expressed as the inverse slope of the regression line through FEV_1_ fall and logged methacholine concentration, lower inverse log slope indicating increased BHR^26^. Using this continuous outcome it was possible to use data from all participants and to maximize power to assess the relationship between fetal growth and BHR using linear regression. The following potential confounders were identified *a priori*: maternal age, body mass index, height, smoking in pregnancy, education, parity, history of asthma, eczema, rhinitis or atopy; paternal history of asthma, eczema or rhinitis; child's gender and parental social class. A forward stepwise multivariate model was built including all variables associated at p ≤ 0.1. Size and growth velocity measures were standardized and outcomes were expressed as change in BHR per SD change in predictor. Stata®11 (Stata Corp., College Station, TX) was used for all analyses. An online supplement provides additional detail.

## Author Contributions

J.W.H., C.T., J.S.A.L., K.M.G., H.M.I., C.C., S.M.R. and G.R. designed research; K.C.P., J.S.A.L., C.T., E.R.T., S.A.C., H.A.W., S.W., S.A.C., P.H.M.K. and S.A.D. conducted research; H.M.I., S.A.D., C.T. and K.C.P. analysed data; K.C.P., C.T., S.A.C. and J.W.H. wrote the paper; J.W.H. and G.R. had primary responsibility for final content. All authors read and approved the final manuscript.

## Additional information

**Funding** This work within the Southampton Women's Survey has been funded by the Medical Research Council, University of Southampton, The Gerald Kerkut Charitable Trust, British Heart Foundation, and the Food Standards Agency (contract no N05071). The research is supported by infrastructure provided by the NIHR Southampton Respiratory Biomedical Research Unit, the NIHR Southampton Biomedical Research Centre and the NIHR Wellcome Trust Clinical Research Facility. Dr Katharine Pike was supported by a grant from the British Lung Foundation. Dr Shelley Davis was supported by a Medical Research Council Respiratory Capacity Building PhD Studentship.

## Supplementary Material

Supplementary Information supplementary data

## Figures and Tables

**Table 1:** Linear regression for fetal and infant growth variables and BHR

	Unadjusted analysis				Adjusted analysis			
	Beta	95% CI	P-value	n	Beta	95% CI	P-value	n
Birthweight	−0.040	−0.400 to 0.319	0.83	225	−0.051	−0.408 to 0.307	0.78	224
Head circumference at birth	−0.001	−0.362 to 0.359	0.99	225	−0.017	−0.376 to 0.341	0.92	224
Abdominal circumference at birth	0.108	−0.261 to 0.478	0.56	225	0.134	−0.239 to 0.506	0.48	221
Head circumference growth between 11 and 19 weeks' gestation	−0.152	−0.678 to 0.374	0.57	114	−0.224	−0.726 to 0.279	0.38	113
Abdominal circumference growth between 11 and 19 weeks' gestation	0.490	−0.138 to 1.119	0.13	110	0.643	0.038 to 1.247	**0.037**	109
Head circumference growth between 19 and 34 weeks' gestation	−0.066	−0.410 to 0.278	0.71	216	−0.050	−0.393 to 0.293	0.77	213
Abdominal circumference growth between 19 and 34 weeks' gestation	0.131	−0.199 to 0.461	0.43	224	0.246	−0.095 to 0.584	0.15	221

Measurements of circumferential growth were calculated as the conditional change in the relevant circumference between either 11 and 19 or 19 and 34 weeks' gestation conditional on either 11 or 19 week circumference respectively. Measurements were corrected for regression to the mean and exact age at measurement Not all women were scanned at each time point and positioning of the fetus occasionally precluded some measurements. If identified as a likely confounder in the multivariate model, the following were adjusted for: maternal history of asthma, eczema, rhinitis or atopy; paternal history of asthma, eczema or rhinitis; maternal age, body mass index, height, smoking in pregnancy, educational achievement and parity; child's gender and parental social class.
