# Supplementary material for: Prenatal development is linked to bronchial reactivity: epidemiological and animal model evidence
Source: Sci Rep. 2014 Apr 17;4:4705. doi: 10.1038/srep04705 (PMC3989559; doi:10.1038/srep04705)
Supplement: Supplementary Information — supplementary data [file srep04705-s1.doc]

Prenatal development is linked to bronchial reactivity: epidemiological and animal model evidence

Katharine C Pike MRCPCH PhD^1,3*^, Shelley A Davis PhD^1,2*^, Sam A Collins^2^, Jane SA Lucas FRCPCH PhD^1,3^, Hazel M Inskip PhD^2,5^, Susan J Wilson PhD^1^, Elin R Thomas^2^, Harris A Wain^2^, Piia HM Keskiväli-Bond BSc^2^, Cyrus Cooper FMedSci^2,4,5^, Keith M Godfrey FRCP^2,4,5^, Christopher Torrens PhD^2^, Graham Roberts MRCPCH DM^1,2,3^,**^§^**, John W Holloway PhD^1,2^,**^§^**,

^1^Clinical and Experimental Sciences Academic Unit, University of Southampton Faculty of Medicine, Southampton, UK, ^2^Human Developmental and Health Academic Unit, University of Southampton Faculty of Medicine, Southampton, UK, ^3^NIHR Southampton Respiratory Biomedical Research Unit, ^4^NIHR Southampton Biomedical Research Centre, University of Southampton and University Hospital Southampton NHS Foundation Trust, Southampton, UK, ^5^Medical Research Council Lifecourse Epidemiology Unit, University of Southampton, Southampton, UK.

*These authors contributed equally to this work.

**^§^**These authors jointly directed this work.

Supplemental material

**Methods**

*Dietary protocol*

Virgin female Wistar rats (supplied by Harlan, UK) weighing approximately 200-250 g were mated with stud males. Conception was confirmed by the presence of a vaginal plug. Once pregnant, animals were fed either a control (C; 18 % casein) or a protein-restricted diet (PR; 9 % casein) through to delivery. The experimental diet constituents were as previously described by Itoh et al.^1^ Mother and pups were returned to standard laboratory chow postpartum.

Pups were weighed 48 h after birth (to avoid rejection) and litters were culled to eight by cervical dislocation, with equal male and female offspring where possible. The offspring were weaned from their mothers at 21 days of age and then separated into male and female cages. At 35, 75 or 225 days of age male offspring were sacrificed by cervical dislocation and lung tissue was harvested.

*Assessment of bronchoconstriction*

Following post mortem lungs were excised and placed in ice cold (4° C) physiological salt solution (PSS) of the following composition; NaCl, 119; KCl, 4.7; CaCl_2_, 2.5; MgSO_4_, 1.17; NaHCO_3_, 25; KH_2_PO_4_, 1.18; EDTA, 0.026; and D-glucose, 5.5 mM. Segments of bronchi were dissected out, cleaned of surrounding tissue, cut into 2 mm segments and mounted on the wire myograph (Danish Myo Technology A/S, DK). Segments of bronchi were maintained in PSS heated to 37°C and continually gassed with 95% O_2_ and 5% CO_2_.

The segments were stretched to an optimal resting tension equal to 1.5 g and allowed to equilibrate for 1 hour. Functional integrity was tested by the addition of 125 mM KPSS solution (PSS with an equimolar substitution of KCl for NaCl). Bronchoconstriction was assessed by the construction of cumulative concentration-response curves to the acetylcholine mimetic, carbachol (CCh, 1 nM-10 µM) and the thromboxane mimetic, U46619 (1pM-1µM).

*Morphometry*

Following post mortem left lungs were formalin fixed for 24 hours then embedded in Paraffin wax. 5µm thick sections were taken from a random starting point (first full block of paraffin cut that included tissue). Entire lung was cut, with 20 sections per layer and 100 sections removed between layers. One section per layer was H&E stained. Stereology was done using volume fractionation to estimate percentage components of the lung structure as previously described.^2^

*Human Participants*

Participants were mother-child pairs participating in the Southampton Women’s Survey; details of follow-up within this study have been published previously.^3^ Briefly, during 1998 - 2002 women aged 20 - 34 years were recruited and those who became pregnant were followed through pregnancy and during their child’s infancy and childhood. Childhood follow-up visits were conducted at 6, 12, 24 and 36 months and those children aged between their sixth and seventh birthdays during 2006 - 2010 were invited for detailed respiratory follow-up during this period. 951 children attended the respiratory follow-up; of these 246 agreed to undergo a methacholine provocation challenge. Seven of the children who underwent methacholine challenge were born < 35 weeks’ gestation; data from these children were excluded to remove the effects of prematurity upon respiratory development. Parental consent was obtained and ethical approval was granted by the Southampton and South West Hampshire Local Research Ethics Committee (LREC Number 276/97, 307/97, 089/99 and 06/Q1702/104).

*Fetal growth*

Gestational age was determined from last menstrual period and early ultrasound data. Experienced research ultrasonographers used Acuson 128 XP, Aspen and Sequoia ultrasound machines calibrated to 1540 m/s to measure fetal head and abdominal circumferences at 11, 19 and 34 weeks gestation from standardized anatomical landmarks. Research nurses measured head and abdominal circumferences and weight at birth. The method of Royston was used to calculate conditional velocities of prenatal head and abdominal circumference growth, correcting for exact age at measurement and for regression to the mean.^4^

*Bronchial hyperreactivity*

Bronchial hyperreactivity was measured by bronchial provocation challenge, according to ATS/ERS guidelines. Incremental methacholine concentrations (0.06 mg/ml to 16 mg/ml) were delivered using a dosimeter (Koko; PDS Instrumentation; Louisville, USA) and a compressed air driven nebulizer (Sidestream®; Respironics, UK).^5^ Challenges were terminated following a 20% fall in the FEV_1_ or, if this did not occur, following the 16 mg/ml dose. BHR was expressed as the inverse of the slope of the regression line through FEV_1_ drop and logged methacholine concentration such that lower inverse log slope values indicate increased BHR.

Log slope=100/[regression slope of FEV_1_ drop and log_10_(cumulative methacholine dose) + 10]

A constant removes negative values and an inverse transformation ensures the variable is normally distributed.^6^

*Calculations and statistical analysis*

All animal data are expressed as mean ± (S.E.M). Constrictor responses are expressed as the change in raw tension (g). Cumulative CRCs to agonists were analysed by fitting to a four-parameter logistic equation using non-linear regression to obtain the pEC_50_ (effective concentration equal to 50% of maximum) and a maximal response, which were compared by Student’s *t* test (Prism 5.0, GraphPAD software Inc., San Diego, CA, U.S.A.). Significance was accepted if *p*<0.05. At all points the investigator was blinded to the dietary group.

The relationships between fetal growth and the continuous BHR outcome was explored using linear regression. The following potential confounders were identified *a priori:* maternal history of asthma, eczema, rhinitis or atopy; paternal history of asthma, eczema or rhinitis; maternal age, body mass index, height, smoking in pregnancy, educational achievement and parity; child’s gender and parental social class. A multivariate model was built using a forward stepwise method to include all variables associated at the 0.1 level of significance or below. Measures of fetal size and growth velocity were standardized and outcomes were expressed in units of change in BHR per SD change in predictor. Stata® 11 (Stata Corp., College Station, TX) was used for all analyses.

References:

1. Itoh H, Ohshima S, Shumiya S, Sakaguchi E. Development of a diet for long-term raising of f344 rats--relationship between dietary digestible crude protein content and digestible energy content. *Experimental animals / Japanese Association for Laboratory Animal Science* 2002;51:317-326.

2. Howard C & Reed M. Unbiased stereology: Three-dimentional measurment in microscopy. Oxford, United Kingdom: Bios Scientific Publishers; 1998.

3. Inskip HM, Godfrey KM, Robinson SM, Law CM, Barker DJ, Cooper C. Cohort profile: The southampton women's survey. *Int J Epidemiol* 2006;35:42-48.

4. Royston P, Altman DG. Design and analysis of longitudinal studies of fetal size. *Ultrasound Obstet Gynecol* 1995;6:307-312.

5. Crapo RO, Casaburi R, Coates AL, Enright PL, Hankinson JL, Irvin CG, MacIntyre NR, McKay RT, Wanger JS, Anderson SD, Cockcroft DW, Fish JE, Sterk PJ. Guidelines for methacholine and exercise challenge testing-1999. This official statement of the american thoracic society was adopted by the ats board of directors, july 1999. *Am J Respir Crit Care Med* 2000;161:309-329.

6. Chinn S, Arossa WA, Jarvis DL, Luczynska CM, Burney PG. Variation in nebulizer aerosol output and weight output from the mefar dosimeter: Implications for multicentre studies. *Eur Respir J* 1997;10:452-456.

**Table S1**. Comparison of SWS mother-child pairs with 6 year follow-up data with those without but born within the same time period.

|  | | **Mother-child pairs in 6 year follow-up (n=925)** | **Mother-child pairs where child aged 6 during follow-up but child not seen (n=570)** | **P-value** |
| --- | --- | --- | --- | --- |
| **Maternal characteristics** | |  |  |  |
| Age at child’s birth (mean (SD)) | | 30.24 (3.81) | 29.76 (3.75) | 0.017 |
| Primiparous (n (%)) | |  |  |  |
| No | | 486 (52.60) | 349 (61.23) | 0.001 |
| Yes | | 438 (47.40) | 221 (38.77) |  |
| Education attainment  (n (%))^*^ | None | 14 (1.52) | 35 (6.15) | <0.001 |
|  | GCSE D-G | 88 (9.52) | 64 (11.25) |  |
|  | GCSE A*-C | 268 (29.00) | 161 (28.30) |  |
|  | A Level | 269 (29.11) | 170 (29.88) |  |
|  | HND | 67 (7.25) | 39 (6.85) |  |
|  | University degree | 218 (23.59) | 100 (17.57) |  |
| Parents’ social class  (n (%))^†^ | I | 97 (10.60) | 49 (11.95) | 0.028 |
|  | II | 452 (49.40) | 177 (43.17) |  |
|  | III Non-manual | 253 (27.65) | 108 (26.34) |  |
|  | III Manual | 72 (7.87) | 50 (12.20) |  |
|  | IV | 39 (4.26) | 19 (4.63) |  |
|  | V | 2 (0.22) | 7 (1.71) |  |
| Smoked in pregnancy (n (%)) | |  |  |  |
| No | | 771 (85.29) | 428 (77.54) | <0.001 |
| Yes | | 133 (14.71) | 124 (22.46 |  |
| Maternal asthma (n (%)) | |  |  |  |
| No | | 720 (78.60) | 429 (76.20) | 0.281 |
| Yes | | 196 (21.40) | 134 (23.80) |  |
| Maternal childhood eczema (n (%)) | |  |  |  |
| No | | 749 (81.86) | 462 (82.06) | 0.922 |
| Yes | | 166 (18.14) | 101 (17.94) |  |
| Maternal rhinitis (n (%)) | |  |  |  |
| No | | 529 (57.75) | 342 (60.75) | 0.256 |
| Yes | | 387 (42.25) | 221 (39.25) |  |
| Maternal atopy (n (%)) | |  |  |  |
| No | | 431 (52.75) | 232 (57.43) | 0.123 |
| Yes | | 386 (47.25) | 172 (42.57) |  |
| Pre-pregnancy BMI, kg/m^2^  (median, IQR) | | 24.32 (22.04-27.53) | 24.06 (21.80-27.38) | 0.570 |
| Height, cm (mean, SD) | | 163.53 (6.59) | 162.73 (6.00) | 0.020 |
| **Paternal characteristics** | |  |  |  |
| Paternal asthma (n (%)) | |  |  |  |
| No | | 752 (82.73) | 440 (79.85) | 0.169 |
| Yes | | 157 (17.27) | 111 (20.15) |  |
| Paternal childhood eczema (n (%)) | |  |  |  |
| No | | 792 (88.00) | 487 (88.55) | 0755 |
| Yes | | 108 (12.00) | 63 (11.45) |  |
| Paternal rhinitis (n (%)) | |  |  |  |
| No | | 592 (65.70) | 370 (67.03) | 0.604 |
| Yes | | 309 (34.30) | 182 (32.97) |  |
| **Child’s characteristics** | |  |  |  |
| Gender (n (%)) | |  |  |  |
| Male | | 478 (51.68) | 305 (53.70) | 0.448 |
| Female | | 447 (48.32) | 263 (46.30) |  |
| Birth weight, kg (mean (SD)) | | 3480.60 (496.14) | 3474.80 (494.20) | 0.827 |
| Gestational age, weeks (median (IQR)) | | 40.14 (39.14-41.00) | 40.10 (39.14-41.00) | 0.995 |
| Length at birth, cm (mean (SD)) | | 49.93 (2.02) | 49.79 (1.99) | 0.183 |
| Weight age six years, kg (median (IQR)) | | 22.30 (20.40-24.80) | Not measured |  |
| Height age six years, cm (mean (SD)) | | 119.18 (5.20) | Not measured |  |
| Age at testing years, (median (IQR)) | | 6.46 (6.34-6.61) | Not measured |  |

Numbers do not always add to the full column totals due to missing data

Binary outcomes were compared by χ^2^ test, categorical outcomes by a χ^2^ test for trend, and continuous variables using t-tests, after transformation where appropriate, or a rank sum test.

^*^GCSE General certificate of secondary education, high school education (to age 16) graded from G (low) to A* (high), A level Advanced level high school education (to age 18), HND Higher national diploma higher education qualification of slightly lower level than that of a university degree.

^†^Social class graded from V (low) to (I) high according to occupation

**Table S2**. Comparison of SWS mother-child pairs with BHR data with those without but born in the same time period and thus eligible for 6 year follow-up.

|  | | **Mother-child pairs with BHR data (n=239)** | **Mother-child pairs without BHR data (n=1256)** | **P-value** |
| --- | --- | --- | --- | --- |
| **Maternal characteristics** | |  |  |  |
| Age at child’s birth (mean (SD)) | | 30.51 (3.79) | 29.97 (3.79) | 0.04 |
| Primiparous (n (%)) | |  |  |  |
| No | | 151 (63.45) | 684 (54.46) | 0.010 |
| Yes | | 87 (36.55) | 572 (45.54) |  |
| Education attainment  (n (%))^*^ | None | 5 (2.09) | 44 (3.51) | 0.185 |
|  | GCSE D-G | 30 (12.55) | 122 (9.73) |  |
|  | GCSE A*-C | 74 (30.96) | 355 (28.31) |  |
|  | A Level | 74 (30.96) | 365 (29.11) |  |
|  | HND | 11 (4.60) | 95 (7.58) |  |
|  | University degree | 45 (18.83) | 273 (21.77) |  |
| Parents’ social class  (n (%))^†^ | I | 20 (8.37) | 126 (11.60) | 0.146 |
|  | II | 114 (47.70) | 515 (47.42) |  |
|  | III Non-manual | 65 (27.20) | 296 (27.26) |  |
|  | III Manual | 25 (10.46) | 97 (8.93) |  |
|  | IV | 14 (5.86) | 44 (4.05) |  |
|  | V | 1 (0.42) | 8 (0.74) |  |
| Smoked in pregnancy (n (%)) | |  |  |  |
| No | | 198 (84.26) | 1001 (81.98) | 0.403 |
| Yes | | 37 (15.74) | 220 (18.02) |  |
| Maternal asthma (n (%)) | |  |  |  |
| No | | 178 (74.79) | 971 (78.24) | 0.241 |
| Yes | | 60 (25.21) | 270 (21.26) |  |
| Maternal childhood eczema (n (%)) | |  |  |  |
| No | | 198 (83.19) | 1013 (81.69) | 0.582 |
| Yes | | 40 (16.81) | 227 (18.31) |  |
| Maternal rhinitis (n (%)) | |  |  |  |
| No | | 135 (56.72) | 736 (59.31) | 0.458 |
| Yes | | 103 (43.28) | 505 (40.69) |  |
| Maternal atopy (n (%)) | |  |  |  |
| No | | 116 (54.98) | 547 (54.16) | 0.828 |
| Yes | | 95 (45.02) | 463 (45.84) |  |
| Pre-pregnancy BMI, kg/m^2^  (median, IQR) | | 24.73 (22.39-27.57) | 24.11 (21.89-27.49) | 0.185 |
| Height, cm (mean, SD) | | 163.76 (6.56) | 163.12 (6.34) | 0.153 |
| **Paternal characteristics** | |  |  |  |
| Paternal asthma (n (%)) | |  |  |  |
| No | | 199 (84.32) | 993 (81.13) | 0.246 |
| Yes | | 37 (15.68) | 231 (18.87) |  |
| Paternal childhood eczema (n (%)) | |  |  |  |
| No | | 212 (90.99) | 1067 (87.67) | 0.151 |
| Yes | | 21 (9.01) | 150 (12.33) |  |
| Paternal rhinitis (n (%)) | |  |  |  |
| No | | 163 (69.96) | 799 (65.49) | 0.187 |
| Yes | | 70 (30.04) | 421 (34.51) |  |
| **Child’s characteristics** | |  |  |  |
| Gender (n (%)) | |  |  |  |
| Male | | 126 (52.72) | 657 (52.39) | 0.926 |
| Female | | 113 (47.28) | 597 (47.61) |  |
| Birth weight, kg (mean (SD)) | | 3493.85 (474.40) | 3475.47 (499.23) | 0.601 |
| Gestational age, weeks (median (IQR)) | | 40.00 (39.14-40.86) | 40.14 (39.14-41.00) | 0.260 |
| Length at birth, cm (mean (SD)) | | 49.98 (2.04) | 49.86 (2.00) | 0.39 |
| Weight age six years, kg (n (%)) | | 22.3 (20.4-24.9) | Not measured in those not followed up |  |
| Height age six , cm (n (%)) | | 121.16 (5.56) | Not measured in those not followed up |  |
| Age at testing years, (median (IQR)) | | 6.47 (6.35-6.65) | Not measured in those not followed up |  |

Numbers do not always add to the full column totals due to missing data

Binary outcomes were compared by χ^2^ test, categorical outcomes by a χ^2^ test for trend, and continuous variables using t-tests, after transformation where appropriate, or a rank sum test.

^*^GCSE General certificate of secondary education, high school education (to age 16) graded from G (low) to A* (high), A level Advanced level high school education (to age 18), HND Higher national diploma higher education qualification of slightly lower level than that of a university degree.

^†^Social class graded from V (low) to (I) high according to occupation

Table S3. Primers and probes used for mRNA analysis

| Gene |  | Sequence |
| --- | --- | --- |
| β-actin | Forward Primer  Reverse Primer  Probe | 5’- CGTGAAAAGATGACCCAGATCA-3’  5’- CACAGCCTGGATGGCTACGT-3  5’-FAM- TTTGAGACCTTCAACACCCCAGCCAT -TAMRA-3’ |
| Rho A | Forward Primer  Reverse Primer  Probe | 5’- -3’  5’- -3’  5’-FAM- -TAMRA-3’ |
| ROCK1 | Forward Primer  Reverse Primer  Probe | 5’- -3’  5’- -3’  5’-FAM- -TAMRA-3’ |
| ROCK” | Forward Primer  Reverse Primer  Probe | 5’- -3’,  5’- -3’  5’-FAM- -TAMRA-3’ |

**Figure S1: Cumulative addition of CCh to isolated bronchi from 75 day old male offspring from C (ο, n=8) or PR (•, n=8) dams in the absence (A) or presence (B) of the Rho kinase inhibitor Y27632 (10 μM). . * indicates p<0.05 C vs. PR**

**A B**
